# Dynamic Changes in Lung Microbiota of Broilers in Response to Aging and Ammonia Stress

**DOI:** 10.3389/fmicb.2021.696913

**Published:** 2021-08-04

**Authors:** Jian Chen, Ai Jin, Lei Huang, Yan Zhao, Yuwen Li, Haotian Zhang, Xiaojun Yang, Qingzhu Sun

**Affiliations:** College of Animal Science and Technology, Northwest A&F University, Yangling, China

**Keywords:** lung microbiota, broiler development, microbiotal function, large-scale breeding, ammonia stress

## Abstract

Comprehensive microbial analysis has revealed that the lung harbors a complex variety of microbiota, and although the dynamic distribution of the lung microbiota in mice and laying hens of different ages is well established, this distribution has not been clarified in broilers of different ages. Here, we performed 16S rRNA gene sequencing of lung lavage fluid from broilers at 3 (3D), 7 (7D), 14 (14D), 21 (21D), and 35 (35D) days of age to evaluate changes in the composition of their lung microbiota. Upon examination of the composition and function of the broiler lung microbiota, we found that their maturation increased significantly with age. Specifically, the microbiota composition was similar between 7 and 14D and between 21 and 35D. The relative abundance of aerobic bacteria in the broiler lungs gradually increased as the broilers developed, whereas the relative abundance of potentially pathogenic bacteria reached its highest level at 3D. The relative abundance of predicted functions in microbiota was very similar among 3, 7, and 14D, whereas the Glycan Biosynthesis and Metabolism pathway in microbiota was enriched at 21D. These findings suggest that these metabolic pathways play critical roles in shaping broiler microbiota at these age stages. In addition, short-term external ammonia stimulation significantly increased lung inflammation but did not significantly affect the lung microbiota. Taken together, these data reveal the dynamics of age-related changes in the microbiota of broiler lungs and the stability (the significant variation in the microbial composition) of these microbial communities in response to short-term ammonia stress. These findings provide new insights into the development of broiler lung microbiota and serve as a reference for subsequent studies to evaluate disease prevention in broilers subjected to large-scale breeding.

## Introduction

Studies evaluating the colonization of lung microbiota in healthy humans have revealed that the microbial density in these tissues tends to be low at the beginning of their development (Gentle and Lal, 2019). The main bacterial genera present in human lungs include *Prevotella*, *Streptococcus*, *Weberella*, *Fusobacterium*, and *Haemophilus* (Hilty et al., 2010; Dickson et al., 2015; Dickson, 2016). Some studies have shown that changes in the dynamics of the lung microbiota can be linked to specific pathologies, including asthma, cystic fibrosis, lung inflammation, and lung cancer (Gustafson et al., 2010; Huang et al., 2015; Mur et al., 2018; Layeghifard et al., 2019; Li et al., 2019). Bacterial communities in the lungs also change in response to changes in the immune status of these tissues. For example, acute lung injury induces an increase in inflammation, which causes distinct changes in the community composition of the lung microbiota (Poroyko et al., 2015). These findings indicate that lung microorganisms play a vital role in maintaining the health of animal lungs.

Long-term breeding of broilers primarily focuses on increasing the rate of muscle growth while ignoring other organ systems (Escorcia et al., 2020). Excessive muscle growth causes a change in the lung/body ratio of affected animals (Hassanzadeh et al., 2005), resulting in relatively small respiratory systems and increased susceptibility to infection, for example, in broilers. Most viruses invade broilers through their respiratory tract; however, some vaccines in broilers are administered through the nasal route (Glendinning et al., 2017). As such, studying the lung microbiota during development of broilers could be beneficial to the broiler industry. Many mammalian trials have highlighted the significant effects of the lung microbiota on lung diseases (Huang et al., 2015; Mur et al., 2018; Layeghifard et al., 2019; Li et al., 2019). However, few studies have reported the relationship between the lung microbiota and lung development in poultry (Glendinning et al., 2017). Therefore, there is limited information describing the lung microbiota of broilers prior to slaughter as part of the intensive breeding process.

Broiler breeding environments are complex and subject to various external stimuli. Ammonia (NH_3_), the most common external stimulus, can exert acute and chronic effects in humans (Tualeka et al., 2018). Specifically, exposure to external NH_3_ can damage the human and rat airway (Woto-Gaye et al., 1999; Elfsmark et al., 2019; Agren et al., 2021). In the breeding industry, certain concentrations of NH_3_ can impair livestock production and performance (Tao et al., 2019; Wang et al., 2020), and the international permissible level of NH_3_ in poultry houses is 25 ppm (ACGIH, 2010).

The primary goal of this study was to determine changes in composition, maturation, and function of the lung microbiota of healthy broilers at 3 (3D), 7 (7D), 14 (14D), 21 (21D), and 35 (35D) days of age using 16S rRNA gene sequencing. In addition, we evaluated changes in the lung microbiota and inflammatory status following short-term exposure to low concentrations of external NH_3_. Our data show that broiler lung microbiota undergoes regular changes in response to changes in maturity.

## Materials and Methods

### Animals, Experimental Design, and Sampling

All the birds and experimental protocols used in this study were approved by the Institutional Animal Care and Use Committee of the Northwest A&F University (Permit Number: NWAFAC 1008).

First, 100 1-day-old, unvaccinated, healthy male white feather (Arbor Acres) broilers (obtained from Xi’an Dacheng Poultry Industry Co., Ltd.) were placed in broiler cages under controlled temperature and humidity. The temperature of the room was maintained at 33°C for the first week and reduced gradually over the next 4 weeks to 24°C; the daily photoperiod was 20 h light:4 h dark. The broilers were randomly divided into five groups with four replicate cages (five broilers per cage) each group (20 broilers per group). Two broilers per replicate (four replicates, *n* = 8) were randomly elected and sacrificed at 3, 7, 14, 21, and 35D. All the broilers were provided with standard broiler feed and water *ad libitum*. The broilers were observed daily and considered healthy following physical examination. Lung lavage was performed using sterilized precooled 1× phosphate-buffered saline (PBS) following broiler euthanasia *via* cervical dislocation. The amount of PBS was increased with age (2.5 ml per 3D-old broiler, 3 ml per 7D-old broiler, 4.5 ml per 14D-old broiler, 8 ml per 21D-old broiler, and 10 ml per 35D-old broiler). During lavage, each broiler was injected with PBS through the trachea, which was circulated three times. A total of 1.8 ml of lavage fluid was collected and stored at −80°C. All instruments were sterilized, and the lung lavage fluid was collected for 16S rRNA gene sequencing.

Next, 40 1-day-old, unvaccinated, healthy male white feather (Arbor Acres) broilers (obtained from Xi’an Dacheng Poultry Industry Co., Ltd.) were placed in broiler cages at controlled temperature and humidity as mentioned earlier. All the broilers were considered healthy by physical examination and fed as described above. The animals were given the same diet for 21 days, and 16 22-day-old broilers with similar weight were randomly divided into two groups, control (CON) and NH_3_, with eight birds each. The NH_3_ challenge was performed as follows: NH_3_ concentration <1 ppm for CON group and 25 ± 3 ppm for NH_3_ group. After 7 days (days 22–28), lung lavage fluid was collected as described above.

### Hematoxylin and Eosin Staining

After lung lavage, the right lung tissue was harvested and cleaned using normal saline and then divided into two parts. One part was fixed in 4% paraformaldehyde and embedded in paraffin before being sliced to 4-μm-thick sections, which were then deparaffinized and stained using H&E for further analysis using light microscopy (Servicebio, Wuhan, China). The other part was frozen in liquid nitrogen and stored at −80°C.

### RNA Extraction and Quantitative Reverse Transcription Polymerase Chain Reaction Analysis

We used TRIzol reagent (Taraka, Japan) to isolate total host RNA from broiler lung tissues and then synthesized cDNA using Evo M-MLV RT Kit with gDNA Clean for qPCR (Accurate Biotechnology, Hunan, China). Quantitative reverse transcription polymerase chain reaction (qRT-PCR) was performed using SYBR Green dye and primers against β-actin, interleukin (IL)-1β, and IL-10, designed using NCBI Primer-Blast (Table 1). β-Actin was used as the internal reference gene. All values were calculated using the 2^–Δ^ ^Δ^ ^*CT*^ method.

**TABLE 1:** Primer Sequences for qRT-PCR.

**Primers**	**Sequences (5′–3′)**	**Bases**	**Product length (bp)**
β-Actin	F: AATCAAGATCATTGCCCCACCT	22	173
	R: TGGGTGTTGGTAACAGTCCG	20	
IL-1β	F: TGCCTGCAGAAGAAGCCTCG	20	204
	R: GACGGGCTCAAAAACCTCCT	20	
IL-10	F: GGAGCTGAGGGTGAAGTTTG	20	147
	R: TAGAAGCGCAGCATCTCTGA	20	

*F, forward; IL, interleukin; R, reverse.*

### DNA Extraction and 16S rRNA Gene Amplification

Total DNA was extracted from each sample using EZNA ®Stool DNA Kit (D4015, Omega, Inc., United States) following the instruction manual. The reagents, which are designed to recover DNA from trace amounts of sample, have been shown to be effective in preparing DNA from most bacteria. The total DNA from each sample was then eluted in 50 μl of elution buffer and stored at −80°C until further analysis.

The 5′ ends of the primers were tagged with specific barcodes, and sequencing was performed using universal primers. The reaction was performed in a total volume of 25 μl composed of 12.5 μl PCR Premix, 2.5 μl of each primer, 50 ng of template DNA, and PCR-grade water. V3–V4 primers 341F (5′-CCTACGGGNGGCWGCAG-3′) and 805R (5′-GACTACHVGGGTATCTAATCC-3′) were used to amplify the V3–V4 region of the 16S rRNA gene *via* PCR as described above. The PCR conditions were as follows: 98°C for 30 s; 30 cycles of 98°C for 10 s, 54°C for 30 s, and 72°C for 45 s; followed by 72°C for 10 min and holding at 4°C. Next, 2% agarose gel electrophoresis was used to confirm the PCR products. We used ultrapure water throughout this process instead of a sample solution to exclude the possibility of false-positive PCR results being obtained in the negative controls.

The PCR products were purified using AMPure XT beads (Beckman Coulter Genomics, Danvers, MA, United States) and quantified on Qubit (Invitrogen, United States). The amplicon pools were prepared for sequencing, and the size and quantity of the amplicon library were assessed using Agilent 2100 Bioanalyzer (Agilent, United States) and a Library Quantification Kit for Illumina (Kapa Biosciences, Woburn, MA, United States), respectively. The libraries were sequenced using the NovaSeq PE250 platform, and 16S rRNA gene sequencing was carried out at LC-Bio Technology Co., Ltd., Hang Zhou, Zhejiang Province, China.

### Data Analysis

Paired-end reads were assigned to samples based on their unique barcodes before the barcode and primer sequences were removed using cutadapt (v1.9). These paired-end reads were then merged using FLASH (v1.2.8), and fqtrim (v0.94) was used for quality filtering of the raw reads to obtain high-quality clean tag reads. The specific filtering conditions were as follows. The sequence raw data were quality-filtered using the window method. The default window size of 100 bp was used, and sequence stretches from the window to the 3′ end were trimmed out from the original read when the average window quality value fell below 20 bp. Sequences less than 100 bp after trimming were removed, as were reads containing more than 5% ambiguous bases (N) after trimming. We used the VSEARCH software (v2.3.4) to filter chimeric sequences and then DADA2 for dereplication. This yielded a feature table and feature sequence that were randomly normalized against the same sequence (20,279 bp) and used to calculate alpha and beta diversity. Taxonomic information for each feature was assigned by aligning the representative sequences with those deposited in the SILVA database (v132). The relative abundance of each taxon was computed by normalizing the number of assigned reads to the number of total reads sequenced, and the average abundance of each group was calculated. The Chao1, observed species, Goods coverage, Shannon, and Simpson indices were calculated using QIIME2 and used to reflect alpha diversity. Unifrac-based principal coordinates analysis (PCoA) was also calculated using QIIME2, and the *p*-value for the Unifrac-based PCoA was obtained using analysis of similarities (ANOSIM). All graphs were drawn using R software (v3.5.2). The Blast was used for sequence alignment, and SILVA (v132) was used for feature annotation. Finally, bacterial phenotypes and microbial function were predicted using BugBase and Tax4Fun, respectively. We calculated the microbial abundance of the top 30 genera and used this to identify a correlation between the microbiota. We drew the network diagrams using SparCC; other diagrams were produced using R software (v3.5.2).

### Statistical Analysis

Statistical analyses of the qRT-PCR results were performed using GraphPad Prism 8 (GraphPad, San Diego, CA, United States), and significance of data was determined using two-tailed Student’s *t*-test. *p* < 0.05 or *p* < 0.01 was considered to indicate statistical significance.

## Results

### Changes in Broiler Lung Microbiota With Aging

The number of DNA sequences in the 29 samples ranged from 49,773 to 81,755, with an average of 72,666 sequences per sample. A total of 2,107,325 high-quality sequences were generated from these 29 samples, indicating that the 16S rRNA gene sequencing results were reliable and appropriate for further analysis.

**FIGURE 1:**
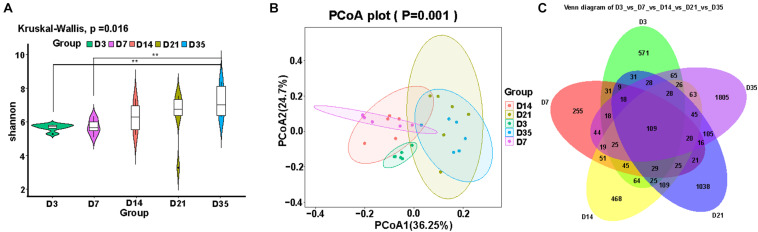
Changes in broiler lung microbiota with age. **(A)** Alpha diversity analysis was performed using the Shannon index. **(B)** Principal coordinates analysis (PCoA) of weighted UniFrac distances as a measure of beta diversity. **(C)** The Venn diagram for feature profiling.

Alpha and beta diversity analyses were used to reflect changes in the lung microbiota. We compared the alpha diversity of the lung microbiota in broilers at 3, 7, 14, 21, and 35D and noted that the Shannon index at 35D was significantly higher than those at 3D (*p* = 0.0020) and 7D (*p* = 0.0040). The Shannon indices at 14 and 21D were not significantly different despite showing an upward trend (Figure 1A). PCoA revealed that the microbial composition was similar between 7 and 14D and between 21 and 35D (Figure 1B). The number of common features in each group was then calculated based on the feature value abundance table and revealed that there were significant increases in feature sequences at 21 and 35D compared with those in the earlier samples (Figure 1C).

### Primary Composition of Broiler Lung Microbiota

**FIGURE 2:**
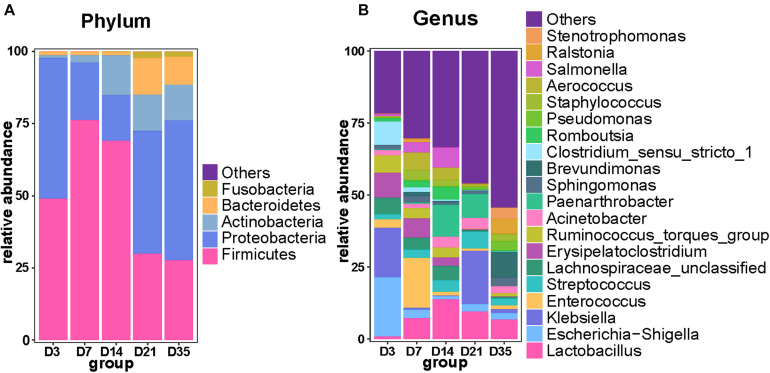
Primary composition of bacteria in broiler lungs. Relative abundance of microbiota among the five groups at the phylum level **(A)** and genus level **(B)**.

As the broilers grew and developed, the abundance of *Firmicutes* decreased, being significantly lower at 35 and 21D than earlier age groups (*p* < 0.01), whereas *Proteobacteria* were significantly more abundant at 35D than at 7D (*p* = 0.0160) and 14D (*p* = 0.0100). Furthermore, *Bacteroidetes* were significantly more abundant at 21D than at 3D (*p* = 0.0006), 7D (*p* = 0.0006), and 14D (*p* = 0.0013). *Actinobacteria* were more abundant at 14D than at 7D (*p* = 0.0109) and 3D (*p* = 0.0025); it reached a relatively stable state after 14D gradually (Figure 2A). Analysis of the top 20 genera revealed that the relative abundance of *Brevundimonas* and *Ralstonia* was significantly increased at 35D compared with that in the other age groups. The relative abundance of *Lactobacillus* was lower at 3D than in any of the other age groups. The 3D group had a much higher abundance of *Escherichia–Shigella* than the other groups, and we also found that the relative abundance of *Klebsiella* was highest at 3 and 21D, whereas that of *Enterococcus* was highest at 7D (Figure 2B).

### Changes in Bacterial Phenotype With Aging

**FIGURE 3:**
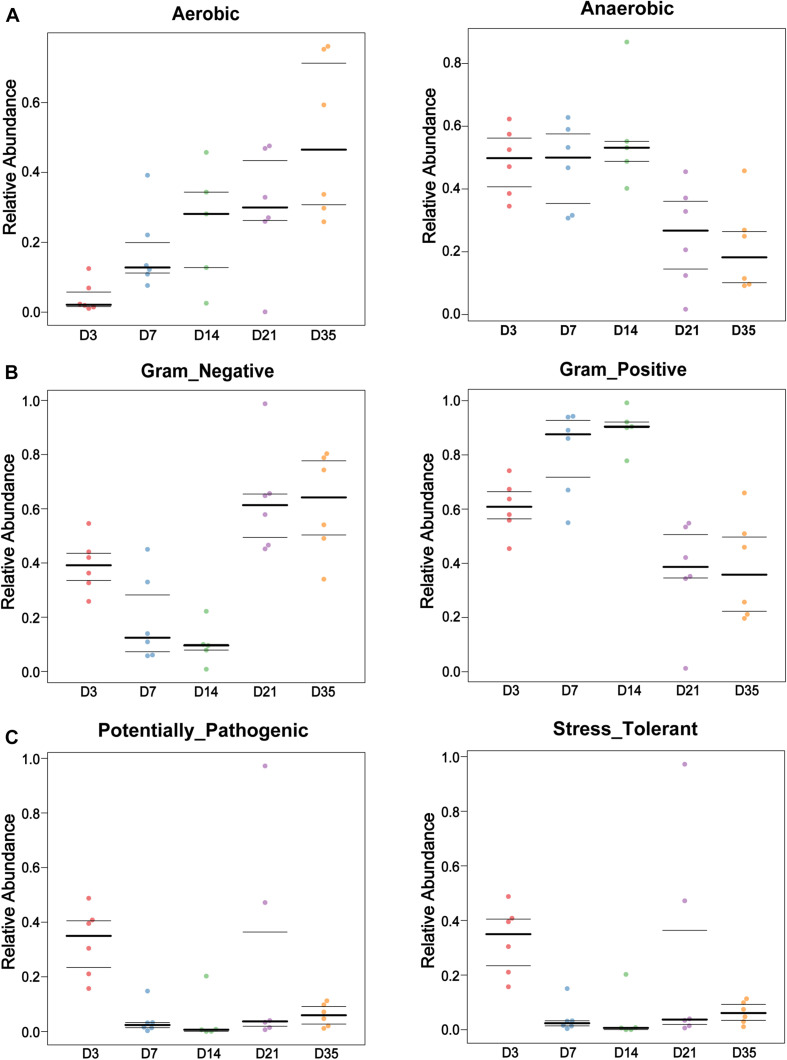
Bacterial phenotypes changed with age. BugBase was used to analyze the lung bacterial phenotypes at different ages in this study. Bar charts showed the phenotypes as Gram-negative and Gram-positive **(A)**, Aerobic and Anaerobic **(B)**, and Potentially Pathogenic and Stress Tolerant **(C)** in each age.

BugBase software was used to analyze bacterial phenotypes. Gram-negative bacteria were significantly more abundant at 35 and 21D than at 14D (*p* < 0.01) and 7D (*p* < 0.01), whereas Gram-positive bacterial abundance displayed a downward trend from 3 to 35D. Furthermore, aerobic bacteria were significantly more abundant at 35D than at 3D (*p* = 0.0022) and 7D (*p* = 0.0087), whereas the number of anaerobic bacteria gradually decreased with increasing age (Figures 3A,B). The number of potentially pathogenic bacteria at 3D was significantly greater than that at 7D (*p* = 0.0022), 14D (*p* = 0.0043), and 35D (*p* = 0.0022), with stress-tolerant microbiota also exhibiting a similar significant difference in abundance between 3 and 7D (*p* = 0.0108), 14D (*p* = 0.0289), and 35D (*p* = 0.0108) (Figure 3C).

### Microbiota Correlation Networks in Different Age Groups

**FIGURE 4:**
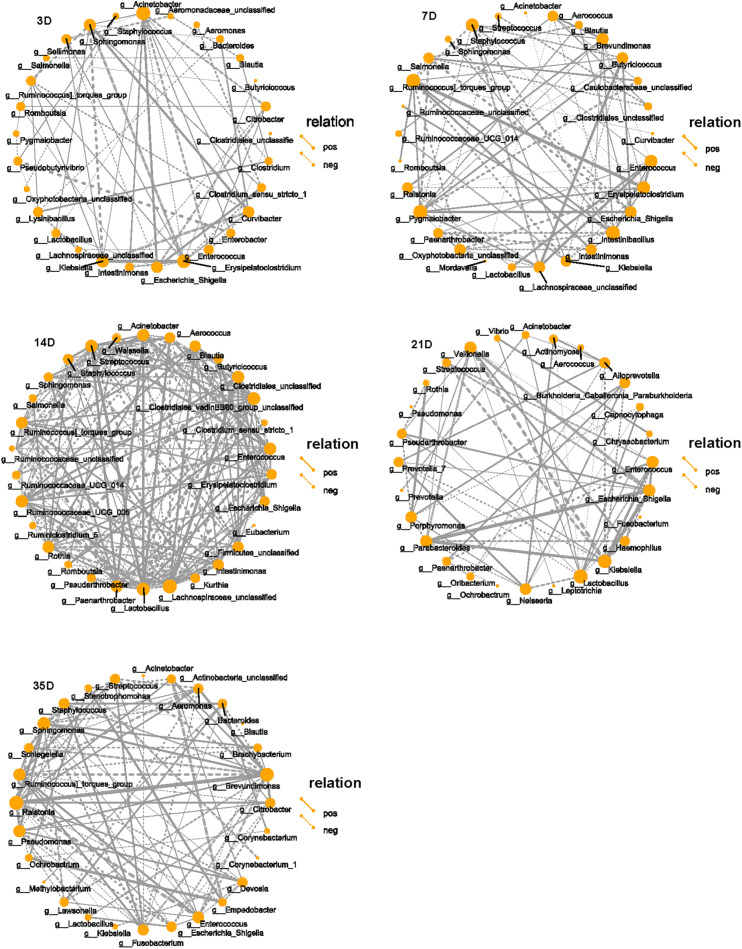
Correlation network of microbiota in each group. SparCC was used to calculate the correlation among the top 30 species in each group and drawing the networks. The solid line indicates the positive correlation, the dashed line indicates the negative correlation, the thickness of the line reflects the strength of the correlation, and the size of the point indicates the number of points that is related to that point. Only showed the relational pair when the correlation coefficient | rho| > 0.4.

The top 30 microbiota in each group were selected for correlation analysis. The taxa playing the most significant roles were observed to be *Klebsiella*, *Erysipelatoclostridium*, *Acinetobacter*, and *Sphingomonas* at 3D and *Brevundimonas*, *Enterococcus*, *Erysipelatoclostridium*, *Intestinibacillus*, *Pygmaiobacter*, *Staphylococcus*, and *Ruminococcin* torques group at 7D. Although there were many interactive associations at 14D, we observed that unclassified *Clostridiales*, unclassified *Lachnospiraceae*, *Lactobacillus*, *Streptococcus*, *Ruminococcaceae UCG-005*, and *Paenarthrobacter* played major roles at this time point. However, *Enterococcus*, *Escherichia–Shigella*, *Klebsiella*, *Lactobacillus*, *Parabacteroides*, and *Veillonella* were dominant at 21D, and *Brevundimonas*, *Ralstonia*, *Stenotrophomonas*, *Enterococcus*, *and Sphingomonas* were dominant at 35D (Figure 4).

### Function of Broiler Lung Microbiota in Different Age Groups

**FIGURE 5:**
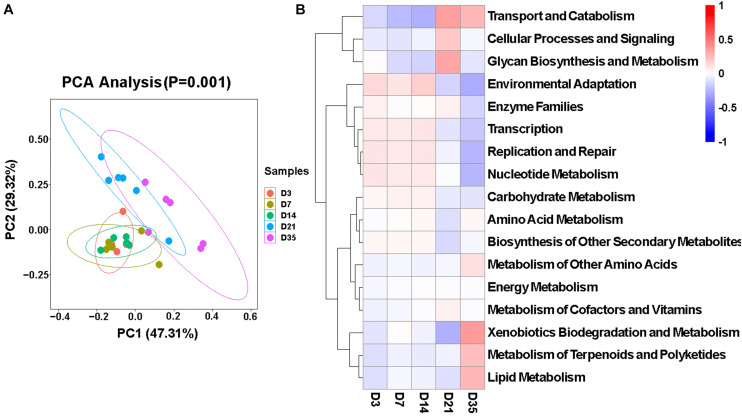
Function of broiler lung microbiota in different age groups. **(A)** Principal component analysis (PCA) plotting for the predicted microbial functions among the five age groups according to the functional annotation and abundance of level 2 Kyoto Encyclopedia of Genes and Genomes (KEGG) pathways using Tax4Fun (*p* = 0.001). **(B)** Heatmap of the different functions identified from the level 2 analysis.

To better understand the microbiota dynamic changes in broilers, the potential functions of lung microbiota in each age group were predicted by Tax4Fun (Asshauer et al., 2015). As was depicted in the principal component analysis (PCA) chart (Figure 5A), potential functions of the microbiota changed as the broilers grew and developed (*p* = 0.001), and they were very similar among 3, 7, and 14D. Consulting the SILVA database (v132), in Level 2, 40 predominant pathways were enriched. Among these, 17 pathways that related to metabolism, cellular processes, and environmental information processing showed significant differences in five age groups (Figure 5B). Of particular interest, the relative abundances of Glycan Biosynthesis and Metabolism significantly increased compared to the other four groups, and at 35D, Xenobiotics Biodegradation and Metabolism, Metabolism of Terpenoids and Polyketides, and Lipid Metabolism were significantly increased.

### Effect of NH_3_ Exposure on Broiler Lung Microbiota

**FIGURE 6:**
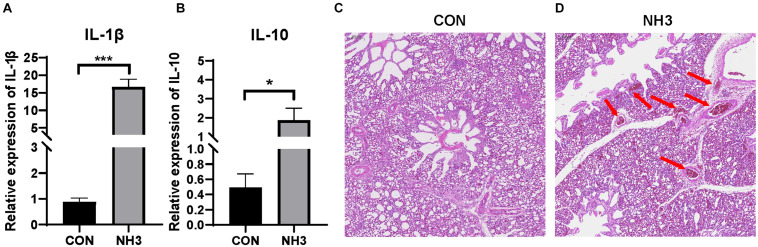
Ammonia exposure caused lung injury. **(A)** Relative expression of interleukin (IL)-1β in the control (CON) (*n* = 6) and ammonia (NH_3_) (*n* = 6) groups. **(B)** Relative expression of IL-10 in the CON and NH_3_ groups. ***p < 0.001 and *p < 0.05 indicate significant differences between the two groups. **(C)** Hematoxylin and eosin-stained sections of the CON group. **(D)** Hematoxylin and eosin-stained sections of the NH_3_ exposure group. The arrow in panel **(D)** indicates local tissue hemorrhage. Scale bar: 100 μm.

Based on the above results, it is obvious that the broiler lung microbiota changes with age. To further investigate whether external stimulation could affect the stability (the significant variation in the microbial composition) of the broiler lung microbiota, we evaluated changes in the microbial communities in response to external NH_3_. Transcriptional profiling of the lung tissues was then performed to evaluate the immune response following 7 days of low-concentration NH_3_ stimulation. We found that the expression of IL-1β (*p* < 0.001) and IL-10 (*p* < 0.05) was significantly elevated in the NH_3_ group in comparison with that in the control (Figures 6A,B). H&E staining of the lung tissues revealed tissues with normal structure without histopathological changes in the CON group (Figure 6C) and local tissue hemorrhage in the NH_3_ group (Figure 6D). These results indicate that short-term NH_3_ induces lung injury in broilers.

**FIGURE 7:**
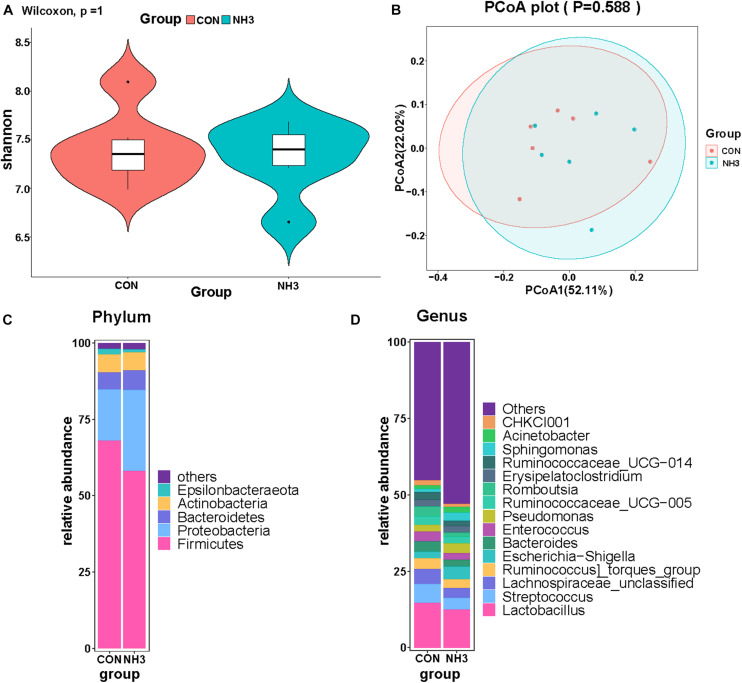
Effect of ammonia exposure on broiler lung microbiota. **(A)** Alpha diversity analysis was performed using the Shannon index. **(B)** Principal coordinates analysis (PCoA) of weighted UniFrac distances as a measure of beta diversity. **(C)** The stack graph of microbiota in major phyla at phylum level. **(D)** The stack graph of microbiota in major phyla at genus level.

We also performed 16S rRNA gene sequencing on lung lavage fluid from the broilers in the CON and NH_3_ groups. The number of DNA sequences for the 12 samples ranged from 59,904 to 80,690, with an average of 75,532 sequences per sample. A total of 906,380 high-quality sequences were generated from the 12 samples. We did not find any significant difference in the alpha diversity of these two groups (Figure 7A). This was mirrored by the lack of changes in the beta diversity distances, measured using PCoA (Figure 7B). Next, we analyzed the broiler lung microbiota composition and visualized the results using a stack graph. We did not observe any significant changes in these parameters between the CON and NH_3_ groups at the phylum and genus levels (Figures 7C,D). Taken together, these data indicate that the composition of the broiler lung microbiota remains stable even in response to short-term treatment with low concentrations of NH_3_.

## Discussion

The lung microbiota has been linked to the development of numerous lung diseases, and recent studies have demonstrated that the composition of the lung microbiota changes with the developmental stages (Kostric et al., 2018). However, research on how broiler lung microbiota changes with aging is scarce. In this study, 16S rRNA gene sequencing was performed on the lung microbiota of broilers at various stages of development. We observed that the maturation of the lung microbiota gradually increased with increasing age and that microbiota composition showed clear age-related clustering.

Stable and diverse lung microbiota confer health benefits to animals. In this study, the alpha diversity of the broiler lung microbiota steadily increased with age and suggested that the maturation of the lung microbiota gradually increased as the birds developed. This result was consistent with those in previous studies that reported that alpha diversity of mouse lung microbiota increased with age before eventually reaching a steady state (Singh et al., 2017; Kostric et al., 2018). As the major respiratory organ, the lung is responsible for communicating with the external environment, allowing outside influences to affect the cardiovascular system and lung microbiota (Kostric et al., 2018). This might be one of the reasons underlying the changes in the alpha diversity of the lung microbiota. Moreover, the composition of the broiler lung microbiota at 3D was different from that in the other age groups, but that between 7 and 14D and between 21 and 35D was similar; these changes in the composition of the lung microbiota were age-dependent. Our results are supported by those in recent studies demonstrating age-related clustering of microbiota in Novogen Brown chickens (Glendinning et al., 2017) and composition of mouse lung microbiota (Kostric et al., 2018).

We also found that aerobic bacteria became more abundant with increasing age, whereas anaerobic bacteria became less abundant in the broiler lungs. This phenomenon may be related to changes in broiler lung ventilation. Furthermore, potentially pathogenic bacteria and stress-tolerant bacteria were more abundant at 3D. Maybe it is because that the broilers were recently exposed to a new environment, stress is put in the lungs and reduces their ability to remove pathogenic bacteria. Subsequently, as lung immunity was gradually established with age, the abundance of potentially pathogenic and stress-resistant bacteria decreased. It is worth noting that the microbiota can also affect local immunity (Olszak et al., 2012; Gollwitzer et al., 2014).

Interestingly, the potential functions of lung microbiota community were predicted using Tax4Fun from 16S rRNA gene sequence data (i.e., functional pathways were inferred from 16S rRNA gene data and not measured directly). Based on these functional predictions, the relative abundances of Glycan Biosynthesis and Metabolism were significantly increased at 21D; this change implied that the microbial metabolism of Glycan in broilers tends to be vigorous at 21 days of age. We also found that at 35 days of age, there was a higher abundance of functions related to Metabolism of Terpenoids and Polyketides. Terpenoids are a very important secondary metabolite in plants, and they display a wide range of biological activities against cancer (Kashyap et al., 2018), inflammation (Gallily et al., 2018), and a variety of bacterial diseases (Zhang and Demain, 2005), and the polyketides have a similar function (Gomes et al., 2013). This seems to imply that the broilers at 35 days of age may be exposed to some health risks.

NH_3_ is one of the best characterized environmental stressors (Zhu et al., 2020). In this study, we used NH_3_ at a concentration of 25 ± 3 ppm to replicate the environmental stress and observed induction of both IL-1β and IL-10 as well as hemorrhage in the lung tissues. IL-1β and IL-10 are significant cytokines involved in regulating inflammatory response (Li et al., 2016; Zhou et al., 2019; Shi et al., 2020). The results revealed that critical concentrations of NH_3_ induced lung inflammation in broilers. Several studies have shown that 25 ppm of NH_3_ may not be safe for broilers, which is in partial agreement with our results (Sa et al., 2018; Zhou et al., 2020). Despite the inflammation, there were no obvious changes in the lung microbiota of the CON and NH_3_ groups when evaluated using 16S rRNA gene sequencing. However, this finding differs from previous results in chickens where the relative abundance of *Escherichia–Shigella* significantly increased upon exposure to NH_3_ at a concentration of 35 ppm (Liu et al., 2020). The possible reason for these results was that the lung microbiota is a downstream effector of lung inflammation, and the inflammation caused by short-term, low-concentration NH_3_ exposure may be insufficient to disrupt lung microbial homeostasis. This view is consistent with that of Scales et al. (2016), who suggested that inflammation can directly and indirectly modulate composition of the microbiota. In addition, differences in sample type could also explain these discrepancies, as the previous studies used lung tissue, not lavage fluid, for 16S rRNA gene sequencing.

## Conclusion

In summary, the study shows that lung microbiota experience changes in abundance, diversity, microbiota phenotypes, and functions throughout the growth and development of white feather (Arbor Acres) broilers. Besides, the low–concentration, short-term, external NH_3_ stimulation does not significantly change the lung microbiota. This study will help inform future broiler lung microbiome strategies, and these strategies may have beneficial effects on the microbiota homeostasis of broiler lung.

## Data Availability Statement

The datasets presented in this study can be found in online repositories. The names of the repository/repositories and accession number(s) can be found below: https://www.ncbi.nlm.nih.gov/, PRJNA728288.

## Ethics Statement

The animal study was reviewed and approved by Institution Animal Care and Use Committee of the Northwest A&F University (Permit Number: NWAFAC 1008).

## Author Contributions

JC, AJ, XY, and QS conceived, designed and performed all experiments, analyzed the data, contributed to the writing of the manuscript, and engaged in extensive scientific discussion on this study and worked on the manuscript. LH, YZ, YL, and HZ performed part of the experiments. All authors have read and approved the manuscript.

## Conflict of Interest

The authors declare that the research was conducted in the absence of any commercial or financial relationships that could be construed as a potential conflict of interest.

## Publisher’s Note

All claims expressed in this article are solely those of the authors and do not necessarily represent those of their affiliated organizations, or those of the publisher, the editors and the reviewers. Any product that may be evaluated in this article, or claim that may be made by its manufacturer, is not guaranteed or endorsed by the publisher.
